# Artisanal Fisheries Research: A Need for Globalization?

**DOI:** 10.1371/journal.pone.0150689

**Published:** 2016-03-04

**Authors:** José Gilmar C. Oliveira Júnior, Luana P. S. Silva, Ana C. M. Malhado, Vandick S. Batista, Nidia N. Fabré, Richard J. Ladle

**Affiliations:** 1 Institute of Biological and Health Sciences (ICBS), Federal University of Alagoas, Maceió, AL, Brazil; 2 School of Geography and the Environment, University of Oxford, Oxford, United Kingdom; Aristotle University of Thessaloniki, GREECE

## Abstract

Given limited funds for research and widespread degradation of ecosystems, environmental scientists should geographically target their studies where they will be most effective. However, in academic areas such as conservation and natural resource management there is often a mismatch between the geographic foci of research effort/funding and research needs. The former frequently being focused in the developed world while the latter is greater in the biodiverse countries of the Global South. Here, we adopt a bibliometric approach to test this hypothesis using research on artisanal fisheries. Such fisheries occur throughout the world, but are especially prominent in developing countries where they are important for supporting local livelihoods, food security and poverty alleviation. Moreover, most artisanal fisheries in the Global South are unregulated and unmonitored and are in urgent need of science-based management to ensure future sustainability. Our results indicate that, as predicted, global research networks and centres of knowledge production are predominantly located in developed countries, indicating a global mismatch between research needs and capacity.

## Introduction

One of the main consequences of limited funding for scientific research is that global knowledge production can show dramatic geographic variations, with research in many areas dominated by scientists based in institutions in the developed world [1]. This is because economically developed countries contain the strongest universities and research centres, can devote more resources to research, and consequently produce more (and higher impact) publications [2–4]. In contrast, countries in the developing world produce lower volumes of research, much of which is published in low impact publications.

Such global inequalities in scientific capacity have significant practical and economic consequences. For example, the populations of many developing countries are still heavily dependent on exploiting natural resources. Effective management these resources should, ideally, be based on the best and most up-to-date science. It follows that a lack of local/regional scientific capacity could lead to information deficits and poor decision-making. Of course, international scientists could theoretically plug these capacity gaps, but even if this was the case they would be unlikley to have the same access to policy-makers and resource managers as their local counterparts [5]. Specifically, local scientists may sit on government bodies/committees, determine the allocation of research funding and, fundamentally, can more effectively communicate research findings to relevant stakeholders in local languages and cultures. In summary, geographic deficits in research capacity can lead to significant mismatches between research effort and research needs at a global scale [6], with serious practical consequences.

Artisanal fisheries is a potential example of geographic deficits in research capacity. Research in this area frequently suffers from data shortfalls which limit the efficacy of policy development and governance in many countries in the developing world [7]. Artisanal fisheries is characterized by simple technology and low capital investment [8]. It occurs all over the world, but is especially prominent in developing countries where it frequently plays a vital role in supporting local livelihoods, food security and poverty alleviation [9–12]. Moreover, about 90 per cent of those dependent on fisheries for their livelihoods live in developing countries [11]. However, artisanal fisheries in these countries are frequently unregulated [13], under intense pressure from growing populations and have, historically, been far less studied than industrial fisheries. Indeed, assessment and management of artisanal fisheries in developing countries has been characterized as “usually inadequate or absent” [14]. In other words, the research needs associated with tropical artisanal fisheries are immense and are predominantly located in developing countries of the Global South. By extension, it is these areas where global research effort should be focused, preferably with knowledge being produced by scientists who are associated with local scientific institutions.

Here, we assess the global production of scientific knowledge in coastal and marine artisanal fisheries with the aims of identifying: i) the geographic structure of research networks and centres of knowledge production, and; ii) geographical patterns and shortfalls in research effort. Our working hypotheses are: i) network connectivity positively influences the quality and the impact of artisanal fisheries research, and; ii) knowledge production for artisanal/small-scale fisheries will be concentrated in major research institutions from the developed world. We tested these hypotheses through a combination of bibliometrics and networks analysis.

## Materials and Methods

Articles were downloaded on 05 November 2014 from the Web of Science Core Collection™ (WoS). The following search strings were applied: ("artisanal fisheries" OR "artisanal fishing") OR ("Small-scale fisheries"). Our methodology was designed to ‘capture’ a representative and largely geographically unbiased sample of articles on artisanal fisheries (see discussion). The search returned 1,127 records.

Records were manually filtered in two steps: i) books and symposium materials were discarded as the focus of this analysis is on peer-reviewed articles; ii) abstracts of all the remaining articles were checked to confirm that the research theme was associated with artisanal fisheries in coastal and marine areas (including estuaries and lagoons). Those that dealt only with industrial fisheries or with continental freshwater fisheries were discarded.

We divided all the valid records into three distinct time subsets: 1973–2004, 2005–2009, 2010–2014. The decision to group all of the older articles into a single subset was made on the basis that there were a very low number of articles published during this period. For each period we identified the number of countries, institutions, authors and journals associated with the articles. To evaluate the spatial and temporal structure of research networks in our dataset, we assessed country level and institutional level co-authorship interactions for each period.

We used citation data to test our hypothesis that connectivity positively influences the quality and impact of the research. Specifically, we used linear regressions to test if connectivity (measured as the number of countries in co-authorship for an article) influenced the impact of the knowledge produced (measured as direct citations accrued by each article). A two-way ANOVA was performed, with countries and number of co-author countries per article as factors, to test for differences in citations per article between these two factors.

We used the Bibexel v.24-03-25 software [15] to perform the network analysis and Pajek v.4. [16] to plot the networks. We used Inkscape v.0.47 (http://www.inkscape.org) to improve the visualization and quality of the network figures and the software R [17] for all the statistical analysis.

## Results

From the 1,127 records generated by our search on the Web of Science (WoS), 661 were considered valid for our analysis. These articles were published in 165 journals. The ten most popular journals for Artisanal/small-scale coastal fisheries sciences were *Fisheries Research* (79 articles), *Marine Policy* (78), *Ocean & Coastal Management* (33), *ICES Journal of Marine Sciences* (17), *Ecology and Society* (16), *Scientia Marina* (13), *Aquatic Living Resources* (12), *Fisheries Management and Ecology* (12), *Boletim do Instituto de Pesca* (11) and *Revista de Biologia Tropical* (10).

**Fig 1:**
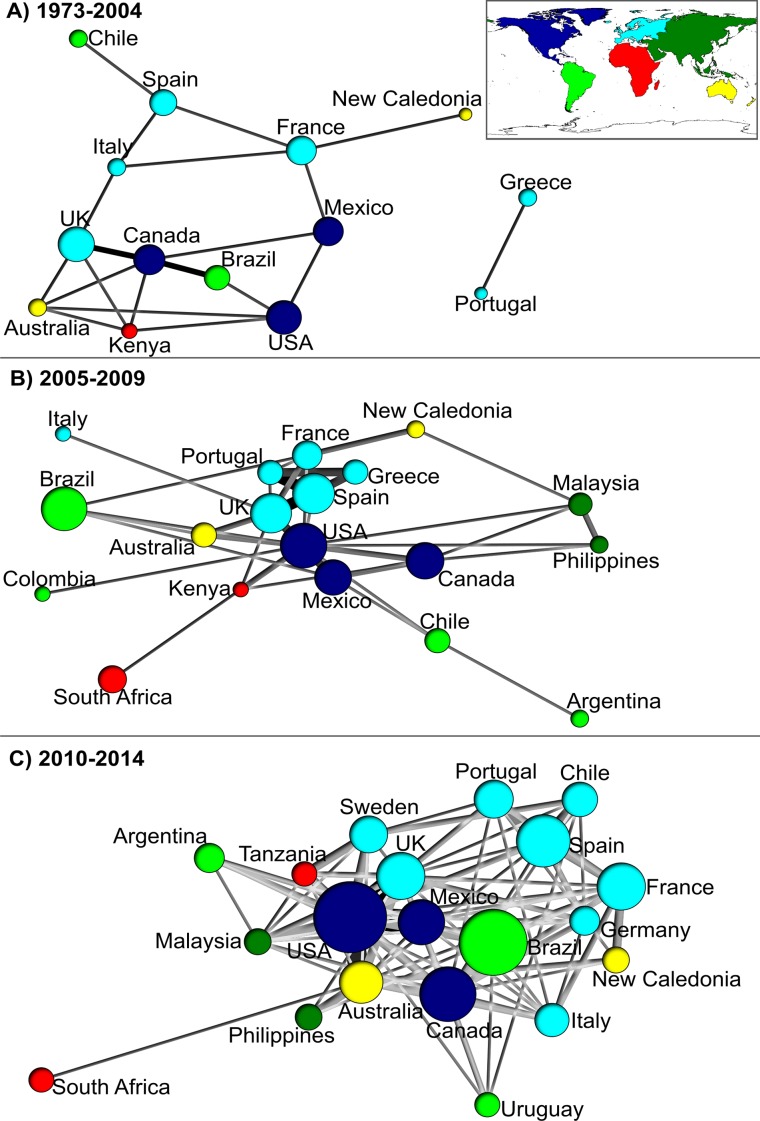
Country level co-authorship networks based on coastal artisanal fisheries articles for the twenty most productive countries based in a sample of 661 articles. Results for the three study periods: A) 1973–2004, B) 2005–2009, C) 2010–2014. Circle size represents the number of articles with at least one author from a particular country. Line weight represents the number of interactions (shared authorships) between two countries. Centrality (betweeness) is determined by the fraction of all directed paths between any two vertices that pass through a node; closeness (distance of one vertex to others) depends on inverse distance to other vertices.

The most productive countries in terms of high quality knowledge production about artisanal/small-scale coastal fisheries were the USA (101 articles), Brazil (85) Canada (65), Spain (63), United Kingdom (63), Mexico (52), France (48), Australia (33), Portugal (27) and Chile (26). The USA was most central in the country level co-authorship networks for the three periods and was also characterized by the greatest number of interactions (Fig 1).

**Fig 2:**
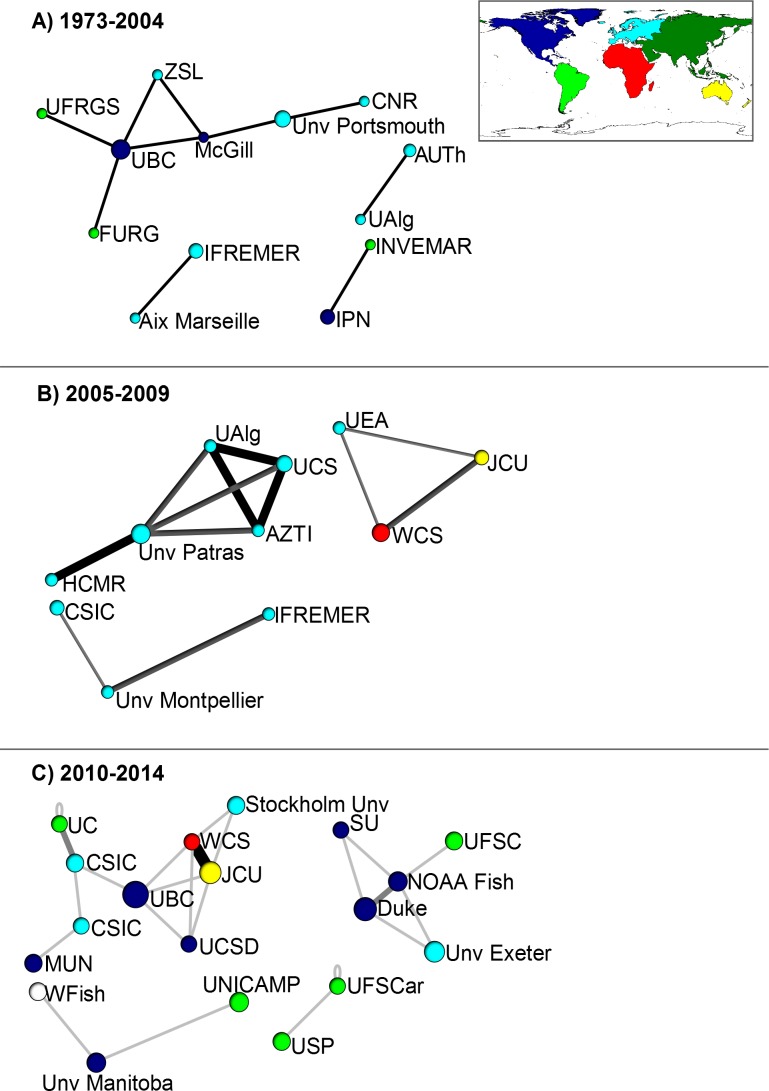
Institutional level co-authorship networks based on coastal artisanal fisheries articles for the twenty most productive countries based in a sample of 661 articles. Results for three study periods: A) 1973–2004, B) 2005–2009, C) 2010–2014. Circle size represents the number of articles with at least one author from a particular country. Line weight represents the number of interactions (shared authorships) between two countries. Centrality (betweeness) is determined by the fraction of all directed paths between any two vertices that pass through a node; closeness (distance of one vertex to others) depends on inverse distance to other vertices. Acronyms: *Aix Marseille*—Aix-Marseille University (France); *AUTh*—Aristotle University of Thessaloniki (Greece); *AZTI*—AZTI-Tecnalia (Spain); *CSIC*—Consejo Superior de Investigaciones Científicas (Spain); *CNR*—Consiglio Nazionale delle Ricerche (Italy); *Duke*—Duke University (USA); *HCMR*—Hellenic Centre for Marine Research (Greece); *IFREMER*—Institut français de recherche pour l'exploitation de la mer (France); *INVEMAR*—Instituto de Investigaciones Marinas y Costeras (Colombia); *IPN*—Instituto Politécnico Nacional (Mexico); *JCU*—James Cook University (Australia); *McGill*—McGill University (Canada); *MUN*—Memorial University of Newfoundland (Canada); *NOOA Fish*—National Oceanic and Atmospheric Administration (USA); *UC*—Pontifica Universidade Catolica do Chile; *Stockholm Unv*—Stockholm University (Sweeden); *UAlg*—Universidade do Algarve (Portugal); *UNICAMP*–Universidade Federal de Campinas (Brazil); *UFSC*—Universidade Federal de Santa Catararina (Brazil); *UFSCar*–Universidade Federal de São Carlos (Brazil); *USP*—Universidade Federal de São Paulo (Brazil); *FURG*—Universidade Federal do Rio Grande (Brazil); *UFRGS*—Universidade Federal do Rio Grande do Sul (Brazil); *UBC*—University British Columbia (Canada); *UCSD*—University of California, San Diego (USA); *UEA*—University of East Anglia (UK); *Unv Exeter*—University of Exeter (England); *Unv Manitoba* -University of Manitoba (Canada); *Unv Montpellier*–University of Montpellier (France); *Unv Patras*—University of Patras (Greece); *WCS*—Wildlife Conservation Society (Kenya); *ZSL*—Zoological Society of London (England); *WFish*–World Fisheries (Worldwide).

The highest production of artisanal fisheries articles was at the following institutions: University British Columbia (30 articles), Ifremer (16), Duke University (14), Wildlife Conservation Society (13), James Cook University (12), Consejo Superior de Investigaciones Científicas (10), WorldFish (10), Pontificia University Catolica Chile (9), University Exeter (9) and NOAA Fisheries (9). Institutions with higher productivity are, as predicted, typically located in Europe and North America, followed by South America, Africa and Oceania (Fig 2).

**Fig 3:**
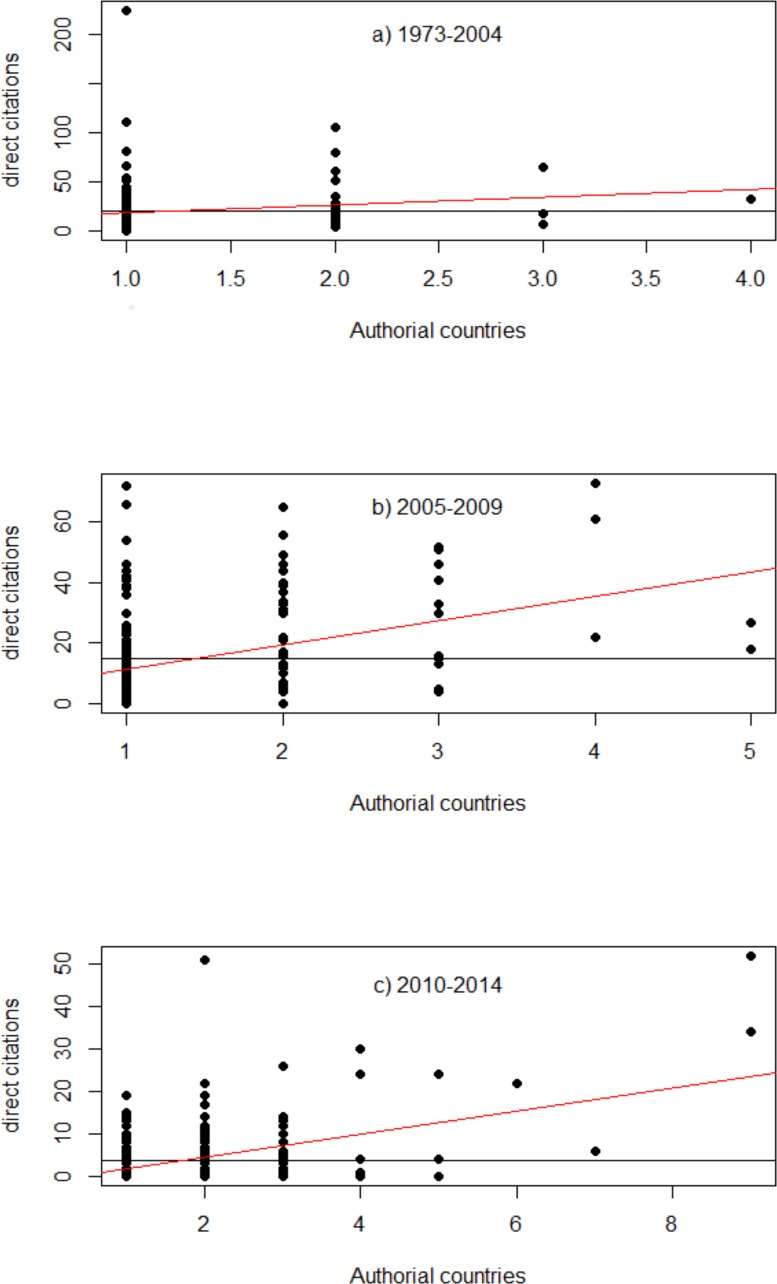
Linear regressions showing the association between connectivity (number of co-authorial countries) and direct citations accrued by each article for the three periods evaluated. The black line represents the average direct citations and the red line represents the linearity between the number of authorial countries and direct citations.

Connectivity and the number of direct citations accrued by an article was significantly correlated for the period 2005–2009 (r^2^ = 0.14, p<0.001) and 2010–2014 (r^2^ = 0.2, p<0.001) (Fig 3) The ANOVA indicated that variations in citations per article are associated with number of co-author countries per article for the periods of 2005–2009 and 2010 and 2014 (p<0.001), but not for 1973–2004 (p>0.05). The variations in citations per article were significantly related to country of origin only for the period of 2005–2014 (p<0.001), but not for the periods of 1973–2004 and 2010–2014 (p≥1.0). The correlation was not significant for 1973–2004 (r^2^ = 0.02, p>0.05).

## Discussion

Artisanal fisheries are vital source of income and food for vast numbers of families in the coastal tropics [7,12]. However, rapidly growing populations and a lack of governance is putting increasing pressure on these poorly known fisheries [11]. We hypothesized that coastal artisanal fisheries research is subject to a geographical mismatch: while research needs are greatest in the developing world tropics, knowledge production, major research institutions and research networks are predominantly based in the developed world. This was largely supported by our data, with both the historical and current production of knowledge about artisanal fisheries aggregated in institutions from developed countries such as the United States of America, Canada, United Kingdom, Australia and France.

The exception to this pattern of developed world dominance of coastal artisanal fisheries research appears to be Brazil, which accounts for a significant proportion of global production in this research area. However, Brazilian-based scientists had surprisingly few interactions with scientists situated other countries, despite the large number of scientific articles produced. These results are consistent with certain aspects of the culture of science in Brazil: although the country is one of the largest producers of environmental research [18], it also has one of the most insular scientific cultures in terms of adjusted levels of national self-citation [19]. There are many potential explanations for such insularity, including a policy focus on local/applied issues, an inward looking scientific culture or a lack of appropriate post-graduate training [19]. It should be noted that international collaboration directly influences journal placement, visibility and the citations [20,21] and, by extension, scientists in more insular scientific cultures may publish more in low impact national journals. Interestingly, the Brazilian government has recently invested heavily in a programme of research visits for its academics (known as the Science without Borders [*Ciência sem Fronteiras*] programme) with the aim of building international research collaborations and increasing national research capacity [22].

European countries were particularly prominent in country and institutional level research networks, possibly because of strong economic relations and a shared interest in fisheries stocks in the Mediterranean Sea– 21 European countries have coastal areas in this region. Moreover, the Mediterranean Sea is a hotspot of biodiversity [23,24] where scientific and management advice is implemented by national and regional entities as FAO, European Community (EC) and others [25]. The strong representation of European countries in artisanal fisheries research networks may also be a consequence of the European Union policy in collecting data to evaluate the fisheries states in Europe [24].

In contrast, Australia and New Caledonia though geographically close, only co-produced knowledge on artisanal fisheries in the final period. Interestingly, New Caledonia appears to have stronger scientific relations with France than Australia–possibly due to long-standing colonial ties based on shared language and culture.

The high representation of the USA, Canada and United Kingdom and their extensive collaborations with other countries is interesting given that the practice of coastal artisanal fisheries is weak in these high-income temperate countries. Of course, some of these linkages may be a consequence of so called Diaspora Knowledge Networks (DKNs), whereby émigré researchers exploit pre-existing research linkages and collaborations in their countries of origin [26]. Alternatively, such networks may have formed around traditional lines, through participation in scientific events such as workshops, seminars, conferences and congresses [27].

Our data also confirm that participation in research networks is associated with increasing quality of research as measured by direct citations. This is well supported both conceptually and empirically [28]. Specifically, Katz and Hick’s widely cited analysis suggested that whereas collaborating with a scientist from the same country could raise citations accrued by 0.75 citations per annum, collaborating with a scientist from a foreign institution raises citations by more than twice that value (1.6). It should also be noted that collaboration with highly productive scientists tends to increase personal productivity (e.g. [29]), whereas collaboration with less productive researchers usually decreases it [30]–this may act as a disincentive for some scientists in western institutions to collaborate with colleagues from less prestigious organizations elsewhere in the world [31,32].

There are several potential reasons why many large tropical countries are poorly represented in global research networks studying artisanal fisheries. First, the dominance of developed countries is a characteristic of scientific research in general, regardless of the geographic focus of the research [4]. A good example is Amazonian research, which has been historically dominated by researchers from North America and European institutions [5]. Second, global research needs do not necessarily map onto research action. These necessities have to be recognized, funds need to be allocated, and research needs to be completed and turned into worldwide useful products (high impact scientific papers). Many developing countries lack research funds in many research areas and may therefore be heavily dependent upon decisions made by international funding organizations. Finally, scientists from tropical developing countries may have a greater focus on local or regional issues [20] and may therefore find it difficult to publish their work in international journals.

In summary, artisanal fisheries research provides a clear demonstration of the global mismatch between research needs and research capacity that is common to many research areas in environmental sciences. To better understand the limitations and barriers to addressing this mismatch it would be necessary to identify a flow of information between (national and international) scientists and policy-makers and how this, in turn, influences research at the local level. Given that the country’s most in need of research to inform policy and management decisions are often those most dependent upon foreign funding and expertise, urgent action is clearly needed to increase international collaborations and local capacity raising. Such action will require strategies to strengthen local institutions, invest in local talent, facilitate responsible research by foreigners and to continue monitoring the volume and geographic distribution of scientific publications in this critical and understudied area of environmental research.

Taking a longer term perspective, mismatches between research need and research effort may eventually start to decline due to external factors. There have been huge increases in joint international research since the turn of the Century [33], partly due to the democratizing influence of the world-wide web. These technological changes have had a particularly strong and positive impact on scientists from the developing world, facilitating collaborations and allowing allowed swift sharing of data and methodologies [34].

## Supporting Information

S1 Text Artisanal Fisheries Research a Need for Globalization. (DOCX)

## References

[1] Schott T. World science: Globalization of institutions and participation. Sci Technol Human Values. 1993;18: 196–208.

[2] King DA. The scientific impact of China. Scientometrics. 2004;63: 411–412. 10.1007/s11192-005-0220-4

[3] May RM. The Scientific Wealth of Nations. Science (80-). 1997;275: 793–796.

[4] Smith MJ, Weinberger C, Bruna EM, Allesina S. The Scientific Impact of Nations: Journal Placement and Citation Performance. PLoS One. Public Library of Science; 2014;9: e109195 10.1371/journal.pone.0109195 25296039 PMC4189927

[5] Malhado ACM, de Azevedo RSD, Todd PA, Santos AMC, Fabré NN, Batista VS, et al Geographic and Temporal Trends in Amazonian Knowledge Production. Biotropica. 2014;46: 6–13. 10.1111/btp.12079

[6] Fisher R, Radford BT, Knowlton N, Brainard RE. Global mismatch between research effort and conservation needs of tropical coral reefs. Conserv Lett. 2011;

[7] Batista V da S, Fabré NN, Malhado ACM, Ladle RJ. Tropical Artisanal Coastal Fisheries: Challenges and Future Directions Tropical Artisanal Coastal Fisheries: Rev Fish Sci Aquac. 2014;22: 1–14. 10.1080/10641262.2013.822463

[8] Hawkins JP, Roberts CM. Effects of Artisanal Fishing on Caribbean Coral Reefs. Conserv Biol. 2004;18: 215–226. 10.1111/j.1523-1739.2004.00328.x

[9] Allison EH, Ellis F. The livelihoods approach and management of small-scale fisheries. Mar Policy. 2001;25: 377–388.

[10] Béné C. When fishery rhymes with poverty: A first step beyond the old paradigm on poverty. World Dev. 2003;31: 949–975. 10.1016/S0305-750X(03)00045-7

[11] Béné C, Macfadyen G, Allison EH. Increasing the contribution of small-scale fisheries to poverty alleviation and food security Rome: FAO; 2007.

[12] Béné C, Hersoug B, Allison EH. Not by rent alone: analyzing the pro-poor functions of small-scale fisheries in developing countries. Dev Policy Rev. 2010;28: 325–358. 10.1111/j.1467-7679.2010.00486.x

[13] Ruddle K, Hickey FR. Accounting for the mismanagement of tropical nearshore fisheries. Environ Dev Sustain. 2008;10: 565–589. 10.1007/s10668-008-9152-5

[14] Andrew NL, Béné C, Hall SJ, Allison EH, Heck S, Ratner BD. Diagnosis and management of small-scale fisheries in developing countries. Fish Fish. 2007;8: 227–240. 10.1111/j.1467-2679.2007.00252.x

[15] Persson O, Danell R, Wiborg Schneider J. How to use Bibexcel for various types of bibliometric analysis In: Åström IF, Danell R, Larsen B, Schneider JW, editors. Celebrating Scholarly Communication Studies: A Festschrift for Olle Persson at his 60th Birthday. International Society forScientometrics and Informetrics; 2009 pp. 9–24.

[16] Batagelj V, Mrvar A. Pajek—Analysis and Visualization of Large Networks In: Jünger M, Mutzel P, editors. Graph Drawing Software. Berlin: Springer; 2003 pp. 77–103.

[17] R Core Team. R: A language and environment for statistical computing Vienna, Austria: the R Foundation for Statistical Computing; 2013. 3-900051-07-0

[18] Regalado A. Brazilian Science : Riding a Gusher. Science (80-). 2004;330: 1306–1312. 10.1126/science.330.6009.1306 21127226

[19] Ladle RJ, Todd P a., Malhado ACM. Assessing insularity in global science. Scientometrics. 2012;93: 745–750. 10.1007/s11192-012-0703-z

[20] Collazo-Reyes F. Growth of the number of indexed journals of Latin America and the Caribbean: the effect on the impact of each country. Scientometrics. 2014;98: 197–209.

[21] J G, R A, Editors. Russell JM, Ainsworth S. Mapping S&T Collaboration between Latin America and Europe: Bibliometric Analysis of Co-authorships (1984–2007). In: Collaboration between Latin America and Europe: Bibliometric Analysis of Co-authorships (1984–2007). ditions des Archives Contemporaines. Paris; 2014.

[22] Vasconcelos SMR, Steneck NH, Anderson M, Masuda H, Palacios M, Pinto JCS, et al The new geography of scientific collaborations. EMBO Rep. Nature Publishing Group; 2012;13: 404–407. 10.1038/embor.2012.51 22491030 PMC3343361

[23] Bianchi N, Morri C. Marine biodiversity of the Mediterranean Sea: situation, problems and prospects for future research. Mar Pollut Bull. 2000;40: 367–376. 10.1016/S0025-326X(00)00027-8

[24] Coll M, Piroddi C, Steenbeek J, Kaschner K, Lasram FBR, Aguzzi J, et al The biodiversity of the Mediterranean Sea: Estimates, patterns, and threats. PLoS One. 2010;5 10.1371/journal.pone.0011842 PMC2914016 20689844

[25] Chevalier C. Governance of the Mediterranean Sea-Outlook for the Legal Regime. Malaga: IUCN-Med; 2005.

[26] Meyer JB, Wattiaux JP. Diaspora Knowledge Networks: Vanishing Doubts and Increasing Evidence. Int J Multicult Soc. 2006;8: 4–24.

[27] Liberman S, Wolf KB. The flow of knowledge: Scientific contacts in formal meetings. Soc Networks. 1997;19: 271–283. 10.1016/S0378-8733(96)00303-6

[28] Katz JS, Hicks D. How much is a collaboration worth? A calibrated bibliometric model. Scientometrics. 1997;40: 541–554. 10.1007/BF02459299

[29] Marmolejo-Leyva R, Perez-Angon MA, Russell JM. Mobility and International Collaboration: Case of the Mexican Scientific Diaspora. PLoS One. Public Library of Science; 2015;10: e0126720 10.1371/journal.pone.0126720 26047501 PMC4457895

[30] Katz JS, Martin BR. What is research collaboration? Res Policy. 1997;26: 1–18. 10.1016/S0048-7333(96)00917-1

[31] Lawani SM. Some bibliometric correlates of quality in scientific research. Scientometrics. 1986;9: 13–25. 10.1007/BF02016604

[32] Lee S, Bozeman B. The Impact of Research Collaboration on Scientific Productivity. Soc Stud Sci. 2005;35: 673–702. 10.1177/0306312705052359

[33] Wagner CS, Leydesdorff L. Network structure, self-organization, and the growth of international collaboration in science. Res Policy. 2005;34: 1608–1618.

[34] Duque RB, Miller BP, Barriga O, Shrum W, Henríquez G. Is Internet Use Associated With Reporting Fewer Problems in Collaboration?: Evidence From the Scientific Community in Chile. Sci Commun. 2012;34: 642–678. 10.1177/1075547011432364

