# Supplementary material for: Artisanal Fisheries Research: A Need for Globalization?
Source: PLoS One. 2016 Mar 4;11(3):e0150689. doi: 10.1371/journal.pone.0150689 (PMC4778908; doi:10.1371/journal.pone.0150689)
Supplement: S1 Text — (DOCX) [file pone.0150689.s001.docx]

**S1 Text. Artisanal Fisheries Research: a Need for Globalization**

*Data sampling*

We added information in a supplementary table to give an overview of the production of knowledge in artisanal fisheries and the indicators (social, environmental and scientific) related to this field of research. *Art. Fish. Res.* is the number of articles produced by each country in our sample collected from web of Science [1]. *Scientific prod.*  is the number of scientific documents produced by the country in 2014 collected from SCImago Journal & Country Rank [2]. IFA is the *Inshore Fishing Area* measured in Km^2^ defined as the coastal the area that extends from shore to either 50 km offshore or to the 200m depth wathever comes first [3]. *MPA Coverage* is the percentage area of territorial waters covered by Marine protected areas in each country [4]. *HDI* is the Human Development Index collected from [5].

Table 1: Countries and indicators related to knowledge production in artisanal fisheries research.

| Country | Art. Fish. Res. | Scientific prod. | IFA | MPA Coverage (%) | HDI |
| --- | --- | --- | --- | --- | --- |
| Argentina | 15 | 11472 | 198.340 | 1,64 | 0.836 |
| Australia | 33 | 77880 | 1.169.359 | 33,24 | 0.935 |
| Bangladesh | 2 | 3360 | 32.477 | 2,54 | 0.57 |
| Barbados | 2 | 112 | 342 | 0,07 | 0.785 |
| Belgium | 3 | 28679 | 2.849 | 55,72 | 0.89 |
| Belize | 2 | 26 | 10.491 | 14,21 | 0.715 |
| Benin | 1 | 363 | 3.248 | - | 0.48 |
| Botswana | 4 | 376 | - | - | 0.698 |
| Brazil | 85 | 59736 | 356.850 | 16,34 | 0.755 |
| Burundi | 1 | 37 | - | - | 0.4 |
| Byelarus | 1 | - | - | - | - |
| Cambodia | 3 | 275 | 22.666 | 0,48 | 0.555 |
| Cameroon | 2 | 1039 | 11.788 | 6,46 | 0.512 |
| Canada | 65 | 88117 | 1.186.323 | 1,35 | 0.913 |
| Chile | 26 | 9679 | 227.721 | 4,75 | 0.832 |
| Colombia | 8 | 6795 | 16.166 | 16,13 | 0.72 |
| Congo | 1 | 405 | 7.256 | 33,50 | 0.591 |
| Costa Rica | 1 | 756 | 16.607 | 15,46 | 0.766 |
| Cote Ivoire | 3 | 314 | 12.270 | 2,01 | 0.462 |
| Croatia | 3 | 5533 | 41.983 | 3,51 | 0.818 |
| Cyprus | 1 | 1649 | 3.107 | 1,32 | 0.85 |
| Denmark | 6 | 22187 | 41.696 | 29,15 | 0.923 |
| Dominican Rep | 1 | 99 | 8.311 | 28,58 | 0.715 |
| Ecuador | 3 | 880 | 27.202 | 75,66 | 0.732 |
| Egypt | 3 | 14196 | 52.338 | 13,14 | 0.69 |
| Eritrea | 3 | 26 | 54.887 | - | 0.391 |
| Estonia | 1 | 2562 | 32.245 | 27,54 | 0.861 |
| Fiji | 2 | 207 | 43.264 | 6,15 | 0.727 |
| Finland | 3 | 17203 | 68.963 | 15,65 | 0.883 |
| France | 48 | 104739 | 92.899 | 58,55 | 0.888 |
| French Guiana | 1 | 72 | 17.368 | - | - |
| Germany | 14 | 149595 | 33.767 | 64,46 | 0.916 |
| Ghana | 3 | 1222 | 20.613 | 1,74 | 0.579 |
| Greece | 18 | 16734 | 72.449 | 6,31 | 0.865 |
| Guadeloupe | 1 | 130 | 2.150 | - | - |
| Guatemala | 2 | 171 | 13.862 | 12,99 | 0.627 |
| Honduras | 2 | 73 | 783.945 | 2,66 | 0.606 |
| India | 2 | 114449 | 225.028 | 1,61 | 0.609 |
| Indonesia | 9 | 5499 | 1.215.466 | 5,81 | 0.684 |
| Ireland | 2 | 11272 | 72.258 | 10,18 | 0.916 |
| Italy | 20 | 93064 | 72.000 | 19,90 | 0.873 |
| Jamaica | 1 | 284 | 13.422 | 4,63 | 0.719 |
| Japan | 6 | 114999 | 284.112 | 5,59 | 0.891 |
| Kenya | 12 | 2067 | 8.282 | 10,52 | 0.548 |
| Madagascar | 3 | 252 | 113.091 | 3,45 | 0.51 |
| Malaysia | 16 | 25330 | 174.971 | 2,28 | 0.779 |
| Malta | 1 | 484 | 4.993 | 0,63 | 0.839 |
| Martinique | 2 | 55 | 1.230 | - | - |
| Mauritania | 2 | 40 | 30.596 | 32,17 | 0.506 |
| Mauritius | 1 | 197 | 10.094 | 0,30 | 0.777 |
| Mexico | 52 | 17709 | 313.461 | 18,86 | 0.756 |
| Mozambique | 4 | 216 | 68.599 | 2,41 | 0.416 |
| Netherlands | 11 | 50732 | 21.629 | 61,82 | 0.922 |
| New Caledonia | 14 | 193 | 36.464 | 25,20 | - |
| New Zealand | 5 | 12455 | 196.577 | 12,45 | 0.913 |
| Nicaragua | 1 | 96 | 53.760 km 2 | 37,74 | 0.631 |
| Nigeria | 4 | 5155 | 36.472 | 0,21 | 0.514 |
| Norway | 8 | 17767 | 112.712 | 2,83 | 0.944 |
| Pakistan | 1 | 10541 | 33.476 | 5,85 | 0.538 |
| Panama | 1 | 436 | 52.039 | 7,41 | 0.78 |
| Papua N Guinea | 2 | 147 | 146.250 | 0,35 | 0.505 |
| Peoples R China | 3 | 452877 | 412.515 | 1,65 | 0.727 |
| Peru | 7 | 1508 | 71.057 | 3,93 | 0.734 |
| Philippines | 15 | 1767 | 249.751 | 2,49 | 0.668 |
| Portugal | 27 | 19911 | 23.807 | 4,10 | 0.83 |
| Reunion | 3 | 52 | 459 | - | - |
| Senegal | 5 | 576 | 18.412 | 14,44 | 0.466 |
| Seychelles | 2 | 38 | 31.750 | 0,85 | 0.772 |
| Sierra Leone | 2 | 66 | 18.301 | 8,63 | 0.413 |
| South Africa | 17 | 17464 | 95.450 | 12,76 | 0.666 |
| Spain | 63 | 78817 | 59.585 | 9,37 | 0.8760.876 |
| Sri Lanka | 2 | 1077 | 31.230 | 1,34 | 0.757 |
| St Vincent | 1 | - | 2.223 | 0,61 | - |
| Sweden | 18 | 33847 | 126.955 | 10,23 | 0.907 |
| Switzerland | 1 | 38308 | - | - | 0.93 |
| Taiwan | 1 | - | 57.496 | - | - |
| Tanzania | 9 | 1134 | 18.987 | 18,21 | 0.521 |
| Thailand | 6 | 12061 | 130.553 | 5,07 | 0.726 |
| Trinid & Tobago | 1 | 387 | 18.717 | 2,98 | 0.772 |
| Tunisia | 3 | 5863 | 44.653 | 2,48 | 0.721 |
| Turkey | 8 | 37095 | 52.269 | 2,70 | 0.761 |
| UK | 63 | 160935 | 271.152 | 16,61 | 0.907 |
| Uruguay | 12 | 1281 | 29.820 | 1,73 | 0.793 |
| USA | 101 | 552690 | 413.060 | 30,40 | 0.915 |
| Vanuatu | 1 | 28 | 8.233 | 0,04 | 0.594 |
| Venezuela | 3 | 1592 | 105.714 | 16,01 | 0.762 |
| Vietnam | 4 | 3519 | 176.223 | 1,73 | 0.666 |
| Zambia | 2 | 377 | - | - | 0.586 |

Legend: *Art. Fish. Res*.: number of artisanal fisheries researches produced by each country; *Scientific prod*.: number of scientific documents produced by the country in 2014; IFA: *Inshore Fishing* Area; MPA coverage: percentage area of territorial waters covered by Marine protected areas in each country; *HDI*.: Human Development Index.

**REFERENCES**

1. Web of Science. Web of Science [Internet]. 2014 [cited 5 Nov 2014]. Available: http://apps.webofknowledge.com/UA_GeneralSearch_input.do?product=UA&search_mode=GeneralSearch&SID=2DRqZCPcsKBuhAJ2Vq8&preferencesSaved=

2. SCImago. SCImago Journal & Country Rank [Internet]. 2007 [cited 31 Dec 2015]. Available: http://www.scimagojr.com/countryrank.php

3. Sea Around Us Project. Sea Around Us [Internet]. 2015 [cited 27 Dec 2015]. Available: http://www.seaaroundus.org/data/#/eez

4. The World Bank. World Development Indicators [Internet]. 2015 [cited 22 Dec 2015]. Available: http://data.worldbank.org/products/wdi

5. United Nations Development Programme. International Human Development Indicators [Internet]. 2015 [cited 23 Dec 2015]. Available: http://hdr.undp.org/en/countries
